# Modeling the next generation sequencing sample processing pipeline for the purposes of classification

**DOI:** 10.1186/1471-2105-14-307

**Published:** 2013-10-11

**Authors:** Noushin Ghaffari, Mohammadmahdi R Yousefi, Charles D Johnson, Ivan Ivanov, Edward R Dougherty

**Affiliations:** 1 AgriLife Genomics and Bioinformatics Services, Texas AgriLife Research, Texas A&M System, College Station, Texas, TX, 77843, USA; 2 Department of Electrical and Computer Engineering, The Ohio State University, Columbus, OH, 43210, USA; 3 Department of Veterinary Physiology and Pharmacology, Texas A&M University, College Station, Texas, TX, 77843, USA; 4 Department of Electrical and Computer Engineering, Texas A&M University, College Station, Texas, TX, 77843, USA; 5 Translational Genomics Research Institute (TGen), 400 North Fifth Street, Suite 1600, Phoenix, AZ, 85004 USA

## Abstract

**Background:**

A key goal of systems biology and translational genomics is to utilize high-throughput measurements of cellular states to develop expression-based classifiers for discriminating among different phenotypes. Recent developments of Next Generation Sequencing (NGS) technologies can facilitate classifier design by providing expression measurements for tens of thousands of genes simultaneously via the abundance of their mRNA transcripts. Because NGS technologies result in a nonlinear transformation of the actual expression distributions, their application can result in data that are less discriminative than would be the actual expression levels themselves, were they directly observable.

**Results:**

Using state-of-the-art distributional modeling for the NGS processing pipeline, this paper studies how that pipeline, via the resulting nonlinear transformation, affects classification and feature selection. The effects of different factors are considered and NGS-based classification is compared to SAGE-based classification and classification directly on the raw expression data, which is represented by a very high-dimensional model previously developed for gene expression. As expected, the nonlinear transformation resulting from NGS processing diminishes classification accuracy; however, owing to a larger number of reads, NGS-based classification outperforms SAGE-based classification.

**Conclusions:**

Having high numbers of reads can mitigate the degradation in classification performance resulting from the effects of NGS technologies. Hence, when performing a RNA-Seq analysis, using the highest possible coverage of the genome is recommended for the purposes of classification.

## Background

In recent years, modern high throughput sequencing technologies have become one of the essential tools in measuring the number of transcripts of each gene in a cell population or even in individual cells. Such information could be used to detect differential gene expression due to different treatment or phenotype. In our case we are interested in using gene-expression measurements to classify phenotypes into one of two classes. The accuracy of classification will depend on the manner in which the phenomena are transformed into data by the measurement technology. We consider the effects of Next-Generation Sequencing (NGS) and Serial Analysis of Gene Expression (SAGE) on gene-expression classification using currently accepted measurement modeling. The accuracy of classification problem has previously been addressed for the LC-MS proteomics pipeline, where state-of-the-art modeling is more refined, the purpose being to characterize the effect of various noise sources on classification accuracy [1].

NGS technology provides a discrete counting measurement for gene-expression levels. In particular, RNA-Seq sequences small RNA fragments (mRNA) to measure gene expression. When a gene is expressed, it produces mRNAs. The RNA-Seq experiment randomly shears and converts the RNA fragments to cDNAs, sequences them, and finally outputs the results in the form of short reads [2,3]. After obtaining those reads, a typical part of a processing pipeline is to map them back to a reference genome to determine the gene-expression levels. The number of reads mapped to a gene on the reference genome defines the count data, which is a discrete measure of the gene-expression levels. Two popular models for statistical representation of the discrete NGS data are the negative binomial [4,5] and Poisson [6]. The negative binomial model is more general because it can mitigate over-dispersion issues associated with the Poisson model; however, with the relatively small number of samples available in most current NGS experiments, it is difficult to accurately estimate the dispersion parameter of the negative binomial model. Therefore, in this study we choose to model the NGS data processing pipeline through the transformation via a Poisson model, for the purposes of phenotype classification.

SAGE technology produces short continuous sequences of nucleotides, called tags. After a SAGE experiment is done, one can measure the expression level of a particular region/gene of interest on the genome by counting the number of tags that map to it. SAGE is very similar to RNA-Seq in nature and in terms of statistical modeling. The SAGE data processing pipeline is traditionally modeled as a Poisson random vector [7,8]. We follow the same approach for synthetically generated SAGE-like data sets.

Our overall methodology is to generate three different types of synthetic data: (1) actual gene expression concentration, called MVN-GC, from a multivariate normal (Gaussian) model formulated to model various aspects of gene expression concentration [9]; (2) Poisson transformed MVN-GC data, called NGS-reads, with specifications that resemble NGS reads; and (3) Poisson transformed MVN-GC data, called SAGE-tags, where the characteristics of the data model SAGE data. The classification results related to these three different types of data sets indicate that MVN-GC misclassification errors are lower compared to data subjected to transformations that produce either NGS-reads or SAGE-tags data. Moreover, classification using RNA-Seq synthetic data outperforms classification using SAGE data when the number of reads is in an acceptable range for an RNA-Seq experiment. The better performance is attributed to the significantly higher genome coverage associated with the RNA-Seq technology.

### Next-generation sequencing technologies

*Next-Generation Sequencing* refers to a class of technologies that sequence millions of short DNA fragments in parallel, with a relatively low cost. The length and number of the reads differ based on the technology. Currently, there are three major commercially available platforms/technologies for NGS: (1) Illumina, (2) Roche, and (3) Life Technologies [10,11]. The underlying chemistry and technique used in each platform is unique and affects the output. In this paper, we focus on Illumina sequencers and use the NGS term to refer to this platform. High-throughput sequencing and NGS are used interchangeably.

The specific application of NGS for RNA sequencing is called RNA-Seq [2], which is a high-throughput measurement of gene-expression levels of thousands of genes simultaneously as represented by discrete expression values for regions of interest on the genome (e.g. genes). NGS has many advantages when compared to the available microarray expression platforms. NGS does not depend on prior knowledge about regions of expression to measure a gene [11], whereas the microarray probes are designed based on known sequences [12]. The background correction, probe design and spot filtering, which are typical for microarray-based technology, are no longer problematic due to the different nature of NGS technology. RNA-Seq enables scientists to discover new splice junctions [13], due to its flexibility and independence of the pre-designed probes. The prediction of absolute copy-number variation (CNV) is another great advantage of RNA-Seq, which allows scientists to identify large segments of deletions/duplications in a genome with respect to another (a reference) genome [14]. Detecting single-nucleotide polymorphisms (SNP) is yet another application of RNA-Seq [3,15]. Furthermore, it has been shown that RNA-Seq can detect spike-in RNA controls with good accuracy and reproducibility [16].

The RNA-Seq process starts with samples that are randomly sheared to generate millions of small RNA fragments. These fragments are then converted to cDNA and the adapter sequences are ligated to their ends. The length of a fragment can vary between 30 bp - 110 bp, approximately. The Illumina system provides *flowcells* for *sequencing by synthesis* and its *reversible terminator chemistry*[2,3,10]. A flowcell is an eight-channel glass and each channel is commonly referred to as a *lane*. The size selected fragments with the adapters attach to the flowcell surface inside the lanes and generate clusters of the same fragment through *bridge amplification*. Following the bending and attaching of both sides of the fragment to the surface, the strand duplicates. This process is repeated many times and results in a *cluster* of fragments. After the cluster generation step, a pool of floating nucleotides is added to the flowcell along with DNA polymerase to incorporate to the single strand fragments in each cluster. Each nucleotide incorporation makes a unique fluorescent label and the images are captured after the addition. Finally, image processing and base calling determine the base at each cycle of the sequencing. Each cluster produces a read whose length equals the number of cycles. Each RNA-Seq experiment produces millions of reads depending on the input RNA material, length of the reads, desired coverage for the reference genome, number of samples per lane, etc. Following the sequencing experiment, the expression levels of the genes are estimated by mapping the reads to the reference genome. There are many algorithms developed for this task, including: ELAND [3], Bowtie [17], BWA [18], MAQ [19], SHRiMP [20], mrFast [14], mrsFAST [21], SOAP [22], etc. After the gene expressions are determined, they can be used in further analysis, such as SNP detection or detecting differentially expressed genes.

The entire RNA-Seq sample processing pipeline from generating reads to calling gene expressions can involve different sources of error, e.g. the basecalling is a probabilistic procedure and the quality scores assigned to each base of the reads are prone to small likelihoods of being wrong. The certainty of reference genomes and mapping algorithms are additional issues that need attention. Thus, the entire RNA-Seq sample processing pipeline should be considered from a probabilistic point of view. In this study, we model the above mentioned errors as a noise term in the model of the sample processing pipeline.

## Discussion

In this section, we consider three specific models for “actual” gene expression data, RNA-Seq count data and SAGE tags data. The models are used to synthetically generate data for the simulation experiments described in this paper. The performances of different classification schemes are analyzed and compared across these three synthetically generated types of data.

A common assumption for modeling of the original mRNA expressions is that they follow a multivariate Gaussian distribution [9,23,24]. Starting with this assumption, we model and generate the RNA-Seq and SAGE data by applying a specific nonlinear Poisson transformation to the mRNA expression model. All data are synthetically generated according to a biologically relevant model to emulate the real experimental situations where the number of features/genes is very large, usually tens of thousands, and only a small number of sample points is available. Knowing the full distributional characteristics of the synthetic data makes it possible to measure the classification performance as described by the respective error rates.

### MVN-GC model

The model proposed in [9] uses a block-based structure on the covariance matrix, which is a standard tool to model groups of interacting variables where there is negligible interaction between the groups. In genomics, genes within a block represent a distinct pathway and are correlated, whereas genes not in the same group are uncorrelated [25,26]. Sample points are drawn randomly and independently from two equally likely classes, 0 and 1, each sharing the same *D* features. There are also *c* equally likely subclasses in class 1 with different parameters for the probability distribution of the features. The subclasses model scenarios typically seen as different stages or subtypes of a cancer. Each sample point in class 1 belongs to one and only one of these subclasses. Features are categorized into two major groups: markers and non-markers. Markers resemble genes associated with a disease or condition related to the disease and they have different class-conditional distributions for the two classes.

**Figure 1:**
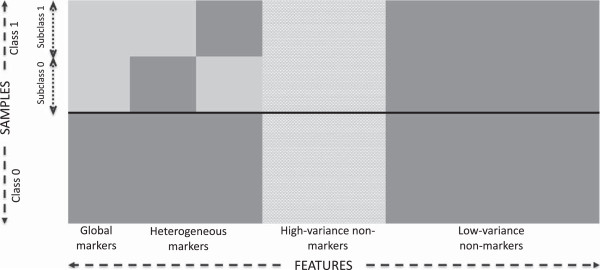
**Multivariate normal distribution model for generating synthetic gene expressions.** This model is proposed by [9].

Markers can be further categorized into two different groups: global and heterogeneous markers. Global markers take on values from *D*_gm_-dimensional Gaussian distributions with parameters $\left(\mu_{0}^{\text{gm}},\Sigma_{0}^{\text{gm}}\right)$ for the sample points from class 0 and $\left(\mu_{1}^{\text{gm}},\Sigma_{1}^{\text{gm}}\right)$ for the points from class 1. Heterogeneous markers, on the other hand, are divided into *c* subgroups of size *D*_hm_, each associated with one of *c* mutually exclusive subclasses within class 1. Therefore, a sample point that belongs to a specific subclass has *D*_hm_ heterogeneous markers distributed as a *D*_hm_-dimensional Gaussian with parameters $\left(\mu_{1}^{\text{hm}},\Sigma_{1}^{\text{hm}}\right)$. The same *D*_hm_ heterogeneous markers for the sample points belonging to other subclasses, as well as points in class 0, follow a different *D*_hm_ -dimensional Gaussian distribution with parameters $\left(\mu_{0}^{\text{hm}},\Sigma_{0}^{\text{hm}}\right)$. We assume that the global and heterogeneous markers have similar structure for the covariance matrices. Therefore, we represent the covariance matrices of these two types of markers by $\Sigma_{0}=\sigma_{0}^{2}\Sigma$ and $\Sigma_{1}=\sigma_{1}^{2}\Sigma$ for class 0 and class 1, respectively, where $\sigma_{0}^{2}$ and $\sigma_{1}^{2}$ can be different. For this structure, we assume that *Σ* has the following block structure:

$$\Sigma =\left[\begin{array}{ccccc} \Sigma_{\rho } & 0 & 0 & \ldots & 0\\ 0 & \Sigma_{\rho } & 0 & \ldots & 0\\ \vdots & \vdots & \ddots & \vdots & \vdots \\ 0 & 0 & \ldots & 0 & \Sigma_{\rho } \end{array}\right]\;,$$

where *Σ*_*ρ*_ is an *l*×*l* matrix, with 1 on the diagonal and *ρ* off the diagonal. Note that the dimension of **Σ** is different for the global and heterogeneous markers. Furthermore, we assume that the mean vectors for the global markers and the heterogeneous markers possess the same structure denoted by *μ*_0_=*m*_0_×(1,1,…,1) and *μ*_1_=*m*_1_×(1,1,…,1) for class 0 and class 1, respectively, where *m*_0_ and *m*_1_ are scalars. The non-markers are also divided into two groups: high-variance and low-variance non-markers. The *D*_hv_ non-markers belonging to the high-variance group are uncorrelated and their distributions are described by $\text{pN}(m_{0},\sigma_{0}^{2})+(1-p)N(m_{1},\sigma_{1}^{2})$, where *m*_0_, *m*_1_, $\sigma_{0}^{2}$ and $\sigma_{1}^{2}$ take values equal to the means and variances of the markers, respectively, and *p* is a random value uniformly distributed over [0, 1]. The *D*_lv_ low-variance non-markers are uncorrelated and have identical one-dimensional Gaussian distributions with parameters $\left(m_{0},\sigma_{0}^{2}\right)$[9,27]. Figure 1 represents the block-based structure of the model.

### NGS-reads and SAGE-tags models

In NGS-type experiments, the gene-expression levels are measured by discrete values providing the number of reads that map to the respective gene. Several statistical models have been proposed for representing NGS data. Those models are based on either negative binomial or Poisson distributions [4-6]. In what follows, we denote the read count for gene *j* for sample point *i* by *X*_*i*,*j*_, where it is assumed that in an NGS experiment each lane has a single biological specimen belonging to either class 0 or class 1. Furthermore, we select a model where the number of reads for each gene is generated from a Poisson distribution with a known parameter. We calculate the expected number of reads (mean of the Poisson distribution) from the generalized linear model [28]:

(1) $$\log(E[\;X_{i,j}|s_{i}])=\log s_{i}+\lambda_{i,j}+\theta_{i,j},$$

where *λ*_*i*,*j*_ is the *j*th gene-expression level in lane *i*. The term *θ*_*i*,*j*_ represents technical effects that might be associated with an experiment. The term log*s*_*i*_ is an offset where *s*_*i*_, referred to as “sequencing depth” in the statistical community [29], denotes a major factor in the transformation from expression levels to read data. It accounts for different total numbers of reads produced by each lane and plays an important role in normalizing the specimens across the flowcell. The trimmed mean of M values (TMM) [30], quantile normalization [28], and median count ratio [4] are three commonly used methods for estimating the sequencing depth. Equation (1) represents the expected value of reads, conditioned on the sequencing depth, based on the linear combination of the factors that affect its value: the depth of sequencing, the gene-expression level and a general noise term. Therefore, it can be used to model the expected number of reads, as the mean of the Poisson distribution in our synthetic data generation pipelines. Rewriting equation (1) yields

(2) $$E[\;X_{i,j}|s_{i}]=s_{i}\exp(\lambda_{i,j}+\theta_{i,j}),$$

indicating that if *λ*_*i*,*j*_ and *θ*_*i*,*j*_ are normally distributed, then exp(*λ*_*i*,*j*_+*θ*_*i*,*j*_) will have a log-normal distribution. Therefore, for a given *s*_*i*_ the mean of *X*_*i*,*j*_ is log-normally distributed. This phenomenon has been previously reported for microarray studies where the means of expression levels are shown to have log-normal distributions [23,31]. Furthermore, we assume that the offset *s*_*i*_ is random and uniformly distributed [29]. Because the term *θ*_*i*,*j*_ represents the unknown technical effects associated with the experimentation, we assume that it follows a Gaussian distribution with zero mean and a variance set by the *coefficient of variation (COV)*:

(3) $$\theta_{i,j}∼N(0,|m_{1}-m_{0}|\text{COV}),$$

COV aims to model the unknown errors that can occur during an/a NGS/SAGE experiment, including basecalling, mapping reads to the reference genome, etc. The term *E*[ *X*_*i*,*j*_|*s*_*i*_] serves as the single parameter of a Poisson distribution that models the NGS/SAGE processes by generating random non-negative integers, as the read counts or tag numbers data, having expected value equal to the right-hand side of equation (2).

To generate synthetic datasets for the purposes of our simulation study we proceed as follows: for a sample point *i* in the experiment, we first randomly generate a number *s*_*i*_ from a uniform distribution, *U* (*α*,*β*), where *α* > 0 and *β* > *α*. Then, a random sample point, *λ*_*i*,*j*_, is drawn from the MVN-GC model and its value is perturbed with *θ*_*i*,*j*_, which is drawn randomly according to its distribution defined in (3). Using (2), the mean of the Poisson distribution is calculated for each sample point *i* and gene *j*, and a single random realization of *X*_*i*,*j*_ is generated. The processes of generating count data for RNA-Seq reads and SAGE tag numbers are very similar, but the total number of reads per NGS run is significantly more than tags for a SAGE experiment. Therefore, we only change *α* and *β* to get the desired number of read counts or tags. We also assume that the SAGE experiments always have a fixed range for the total number of tags, whereas RNA-Seq has a variety of ranges for the total read counts. By having different ranges for the read counts, we can compare the performance of classification resulting from NGS-reads and SAGE-tags models under different experimental settings.

### Classification schemes

The setup for the classification problem is determined by a joint feature-label probability distribution *F*_**X** *Y*_, where $X\in R^{D}$ is a random feature vector and *Y* ∈ {0,1} is the unknown class label of **X**. In the context of genomics, the feature vector is usually the expression levels of many genes and the class labels are different types or stages of disease to which sample points belong to. A classifier rule model is defined as a pair (*Ψ*, Ξ), where *Ψ* is a classification rule, possibly including feature selection, and Ξ is a training-data error estimation rule. In a typical classification task, a random training set $S_{n}=\{(X_{1},Y_{1}),(X_{2},Y_{2}),\ldots ,(X_{n},Y_{n})\}$ is drawn from *F*_**X** *Y*_ and the goal is to design a classifier $\psi_{n}=\Psi (S_{n})$, which takes **X** as the input and outputs a label *Y*. The true classification error of *ψ*_*n*_ is given by *ε*_*n*_ = *P* (*ψ*_*n*_ (**X**) ≠ *Y*). The error estimation rule Ξ provides an error estimate, ${\hat{\varepsilon }}_{n}=\Xi (S_{n})$, for *ψ*_*n*_.

In this study, we consider linear discriminant analysis (LDA), three nearest neighbors (3NN) and radial basis function support vector machine (RBF-SVM) as the classification rules, and report the true error of the classifiers. We implement *t*-test feature selection (as a part of the classification rule) before the classifier design procedure to select *d* ≤ *D* features with highest *t*-scores. The training set with *d* features is then used to design the classifier. The same *d* features are also used for finding the true error of the designed classifier.

LDA is the plug-in rule for the Bayes classifier when the class-conditional densities are multivariate Gaussian with a common covariance matrix [32]. The sample means and pooled sample covariance matrix are estimated from the training data $S_{n}$ and plugged into the discriminant function. If the classes are equally likely, LDA assigns **x** to class 1 if and only if

(4) $$\;{\left(x-{\bar{\mu }}_{1}\right)}^{T}{\hat{\Sigma }}^{-1}(x-{\bar{\mu }}_{1})\leq {\left(x-{\bar{\mu }}_{0}\right)}^{T}{\hat{\Sigma }}^{-1}(x-{\bar{\mu }}_{0}),$$

where ${\bar{\mu }}_{y}$ is the sample mean for class *y* ∈ {0,1}, and $\hat{\Sigma }$ is the pooled sample covariance matrix.

3NN is a special case of *k*NN rule (with *k* = 3), which is a nonparametric classification rule based on the training data. The *k*NN classifier assigns a label, 0 or 1, to a point **x** according to the majority of the labels of the *k* nearest training data points to it. To avoid tied votes in binary classification problems, an odd number is usually chosen for *k*.

A support vector machine finds a maximal margin hyperplane for a given set of training sample points. If it is not possible to linearly separate the data, one can introduce some slack variables in the optimization procedure that allow the mislabeled sample points and solve the dual problem. Alternatively, one can transform the data and project it into a higher-dimensional space, where it becomes linearly separable. The equivalent classifier back in the original feature space will generally be non-linear [33,34]. If a Gaussian radial basis function used as the kernel function, the corresponding classifier is referred to as RBF-SVM.

### Simulation setup and parameters

**Figure 2:**
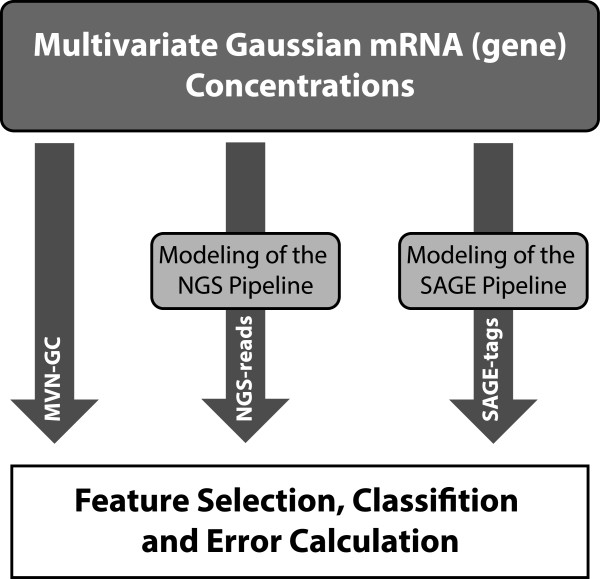
**Three different types of data are generated: (1) MVN-GC; (2) NGS-reads; and (3) SAGE-tags.** Data sets are generated according to the respective statistical models. Then, the data is fed to the feature selection, classification and error estimation module.

Figure 2 presents an overview of the simulations performed in the study. In this section we provide the setup and list of parameters used in the study. The analysis of the results follows in the next section.

To emulate real-world scenarios involving thousands of genes with only a few sample points, we choose *D* = 20,000 as the number of features and *n* ∈ {60, 120, 180} as the number of sample points available for each synthetic feature-label distribution. Because there is no closed form expression to calculate the true error of the designed classifiers, we generate a large independent test sample of size *n*_*t*_ = 3000, with samples divided equally between the two classes. Because the RMS between the true and estimated error when using independent test data is bounded above by $1/2\sqrt{n_{t}}$, this test sample size provides an error-estimate RMS of less than 0.01. Once the training and test data are generated, we normalize the training data so that each feature is zero-mean unit-variance across all sample points in both classes. We also apply the same normalization coefficients from the training data to the test set. The normalized data are then used in the feature selection, classifier design and error calculation. The parameter settings for the MVN-GC model, SAGE-tags and NGS-reads are provided in Table 1.

**Table 1:** MVN-GC, SAGE-tags and NGS-reads models parameters

**Parameters**	**Value**
Feature size *D*	20000
Training sample	60,120,180
size *n*	
Test sample size *n*_*t*_	3000
Class 0 (*m*_0_,*σ*_0_)	(0.0, 0.6)
Class 1 (*m*_1_,*σ*_1_)	(1.0, 0.6)
Correlation *ρ*	0.4, 0.8
Block size *l*	5
Global markers *D*_gm_	10
Subclasses *c*	2
Heterogenous	50
markers per	
subclass *D*_hm_	
High-variance	2000
non-markers *D*_hv_	
Low-variance	17890
non-markers *D*_lv_	
COV	0.05, 0.1
SAGE-tags range	50K-100K
NGS-reads range	1K-50K, 250K-300K, 500K-550K
	5M-5M+50K, 10M-10M+50K, 15M-15M+50K
	25M-25M+50K, 32.5M-32.5M+50K, 40M-40M+50K
	50M-50M+50K, 75M-75M+50K, 100M-100M+50K

As explained in the previous section, the datasets generated from the MVN-GC model are transformed into the NGS-reads and SAGE-tags datasets through equation (2) and Poisson processes. We only need to properly set the parameters COV, *α*, and *β* to get the desired number of read counts or tags. We assume that the parameter COV can take on two values, 0.01 and 0.05, representing two different levels of noise and unknown errors in the experiment. In its current state, RNA-Seq technology can provide different numbers of reads per sample, depending on many factors, such as quality of the sample, the desired coverage, sample multiplexing, etc. In this study, we examine a variety of ranges for RNA-Seq experiments. We start with a very low total number of RNA-Seq reads, which may not match the real-world experiments, however, they are necessary for comparing the SAGE-tags and NGS-reads models with similar coverage. Furthermore, demonstrating the classification results for the NGS-reads model with wide ranges introduces an extra internal variability, which makes interpretations of the results rather difficult. Table 1 lists the NGS-reads ranges we have considered in this study. In a typical SAGE experiment, one expects 50K to 100K tags [35,36]. Using trial and error, we have found that by choosing the parameters *α* = 2.0 and *β* = 3.75 in the distribution of *s*_*i*_, the observed number of tags usually falls within this range. Similarly, the parameters *α* and *β* are chosen to meet the range requirements in the NGS-reads model.

Our goal is to study the performance of the traditional classification schemes on different sources of random samples; thus, we take a Monte-Carlo approach and generate 10,000 random training sets of size *n* from the MVN-GC model, transform them to the corresponding NGS-reads and SAGE-tags samples, and apply the classification schemes to each training sample. By taking such an approach, we aim to compare the classification performance of the three pipelines, in terms of the true error of the deigned classifiers. We also study the effect of NGS-reads and SAGE-tags transformations on the performance of a simple *t*-test biomarker discovery method, where we report the probability that global markers are recovered when *d* ≪ *D* features are selected after the feature-selection step.

## Results

The probability of recovering a certain number of global markers after a *t*-test feature selection can be approximated empirically by the percentage of experiments (out of 10,000 independent experiments) that detect such a number of markers. This probability depends on the size of the training data sample, quality of features, and underlying joint probability distribution of the features. Here, we only show the results for the MVN-GC model, with *d* = 10, in Table 2. Tables 3, 4, 5 and 6 represent the corresponding results for NGS-reads for different combinations of COV and *ρ*: {0.05, 0.4}, {0.05, 0.8}, {0.1, 0.4}, {0.1, 0.8}, respectively. In each table, we also report the corresponding results for the SAGE-tags model in a row with the NGS-reads range of [ 50*K* − 100*K*]. A successful feature selection should identify as many global markers as possible, however the situation is worsened because with small sample sizes the noisy features sometimes may appear to be good among the 20,000 features. As the number of sample points increases, we expect to get better results for the feature selection and this is exactly what we see in Tables 2, 3, 4, 5 and 6. Another important observation in Tables 3, 4, 5 and 6 is the effect of the total number of reads and COV for the NGS-reads models. As the number of reads increases, it is more likely to pick up more global markers, until the number of reads reaches a threshold, where no further improvement is observed. Similarly, for smaller COV the probability of selecting more global markers also increases.

**Table 2:** Percentage of the MVN-GC experiments recovering certain number of global markers after feature selection

**Correlation**	**Sample**	**<8**	**8**	**9**	**10**
	**size**	**markers**	**markers**	**markers**	**markers**
	*n*=60	5.4733	15.8242	40.4508	38.2517
*ρ*=0.4	*n*=120	0.0108	0.2375	6.1483	93.6033
	*n*=180	0.0000	0.0017	0.3367	99.6617
	*n*=60	9.5742	9.7425	22.0408	58.6425
*ρ*=0.8	*n*=120	0.1742	0.4692	2.6925	96.6642
	*n*=180	0.0025	0.0175	0.1933	99.7867

**Table 3:** **Percentage of the NGS-reads experiments recovering certain number of global markers after feature selection for COV=0.05 and** ***ρ*** **=0** ***.*** **4**

**NGS-reads**	**Sample**	**<8**	**8**	**9**	**10**
**range**	**size**	**markers**	**markers**	**markers**	**markers**
	*n*=60	99.64	0.34	0.02	0.00
1K-50K	*n*=120	69.98	20.48	8.64	0.90
	*n*=180	27.84	33.76	31.20	7.20
	*n*=60	77.30	16.54	5.60	0.56
50K-100K (SAGE-tags)	*n*=120	10.03	26.12	44.08	19.77
	*n*=180	0.88	7.53	39.10	52.49
	*n*=60	41.30	32.44	22.00	4.26
250K-300K	*n*=120	2.10	11.82	41.56	44.52
	*n*=180	0.10	1.74	22.88	75.28
	*n*=60	34.54	32.74	26.22	6.50
500K-550K	*n*=120	1.82	11.08	41.56	45.54
	*n*=180	0.10	1.56	20.26	78.08
	*n*=60	30.98	31.36	30.04	7.62
5M-5M+50K	*n*=120	1.24	8.72	37.56	52.48
	*n*=180	0.04	1.20	18.92	79.84
	*n*=60	29.74	32.68	29.16	8.42
10M-10M+50K	*n*=120	1.22	8.28	37.36	53.14
	*n*=180	0.04	1.00	18.84	80.12
	*n*=60	28.96	33.16	29.92	7.96
15M-15M+50K	*n*=120	1.06	8.06	38.38	52.50
	*n*=180	0.06	1.36	18.08	80.50
	*n*=60	28.90	32.36	30.38	8.36
25M-25M+50K	*n*=120	0.98	8.00	40.12	50.90
	*n*=180	0.06	1.14	17.68	81.12
	*n*=60	28.60	32.50	30.42	8.48
32.5M-32.5M+50K	*n*=120	1.10	7.48	39.02	52.40
	*n*=180	0.02	1.02	17.80	81.16
	*n*=60	30.20	31.46	29.40	8.94
40M-40M+50K	*n*=120	1.34	8.50	38.12	52.04
	*n*=180	0.08	1.02	18.62	80.28
	*n*=60	28.84	32.18	30.32	8.66
50M-50M+50K	*n*=120	1.22	8.46	39.02	51.30
	*n*=180	0.04	1.28	17.24	81.44
	*n*=60	29.12	32.50	29.72	8.66
75M-75M+50K	*n*=120	1.08	7.98	39.64	51.30
	*n*=180	0.02	1.16	17.14	81.68
	*n*=60	29.14	32.16	30.84	7.86
100M-100M+50K	*n*=120	1.22	8.78	39.10	50.90
	*n*=180	0.08	1.22	18.32	80.38

**Table 4:** **Percentage of the NGS-reads experiments recovering certain number of global markers after feature selection for COV=0.05 and** ***ρ*** **=0** ***.*** **8**

**NGS-reads**	**Sample**	**<8**	**8**	**9**	**10**
**range**	**size**	**markers**	**markers**	**markers**	**markers**
	*n*=60	99.02	0.80	0.18	0.00
1K-50K	*n*=120	66.16	20.68	11.20	1.96
	*n*=180	28.54	28.38	30.84	12.24
	*n*=60	71.61	17.10	9.28	2.01
50K-100K (SAGE-tags)	*n*=120	12.85	20.14	36.80	30.21
	*n*=180	2.30	7.43	28.67	61.60
	*n*=60	40.94	22.42	24.12	12.52
250K-300K	*n*=120	5.52	9.46	28.58	56.44
	*n*=180	0.70	1.86	14.14	83.30
	*n*=60	34.48	21.02	27.56	16.94
500K-550K	*n*=120	4.84	8.16	24.20	62.80
	*n*=180	0.78	2.16	11.38	85.68
	*n*=60	31.26	20.80	26.64	21.30
5M-5M+50K	*n*=120	3.98	6.62	21.78	67.62
	*n*=180	0.66	2.24	10.72	86.38
	*n*=60	30.04	20.86	26.84	22.26
10M-10M+50K	*n*=120	3.54	7.24	22.06	67.16
	*n*=180	0.88	1.44	9.72	87.96
	*n*=60	30.08	20.68	27.92	21.32
15M-15M+50K	*n*=120	4.32	7.10	22.42	66.16
	*n*=180	0.60	1.72	9.62	88.06
	*n*=60	30.86	19.92	27.00	22.22
25M-25M+50K	*n*=120	3.56	7.46	21.90	67.08
	*n*=180	0.58	1.90	8.90	88.62
	*n*=60	30.50	20.20	27.50	21.80
32.5M-32.5M+50K	*n*=120	3.62	7.00	21.66	67.72
	*n*=180	0.80	1.88	10.22	87.10
	*n*=60	31.14	18.40	28.14	22.32
40M-40M+50K	*n*=120	3.90	7.12	22.44	66.54
	*n*=180	0.60	1.90	10.12	87.38
	*n*=60	30.92	19.34	27.20	22.54
50M-50M+50K	*n*=120	3.58	6.68	22.24	67.50
	*n*=180	0.68	1.66	10.22	87.44
	*n*=60	30.74	19.00	27.92	22.34
75M-75M+50K	*n*=120	3.68	7.64	23.46	65.22
	*n*=180	0.50	1.90	10.44	87.16
	*n*=60	31.06	20.32	26.70	21.92
100M-100M+50K	*n*=120	3.84	6.48	21.30	68.38
	*n*=180	0.86	1.80	9.94	87.40

**Table 5:** **Percentage of the NGS-reads experiments recovering certain number of global markers after feature selection for COV=0.1 and** ***ρ*** **=0** ***.*** **4**

**NGS-reads**	**Sample**	**<8**	**8**	**9**	**10**
**range**	**size**	**markers**	**markers**	**markers**	**markers**
	*n*=60	99.62	0.36	0.02	0.00
1K-50K	*n*=120	70.26	20.64	8.02	1.08
	*n*=180	30.16	32.46	29.80	7.58
	*n*=60	78.67	15.75	5.08	0.50
50K-100K (SAGE-tags)	*n*=120	10.75	24.12	43.45	18.68
	*n*=180	1.01	8.32	40.50	50.17
	*n*=60	44.64	31.70	20.00	3.66
250K-300K	*n*=120	2.60	13.76	44.24	39.40
	*n*=180	0.10	2.08	23.84	73.98
	*n*=60	37.16	32.54	24.48	5.82
500K-550K	*n*=120	1.92	11.42	42.50	44.16
	*n*=180	0.06	1.80	21.98	76.16
	*n*=60	32.36	31.70	28.84	7.10
5M-5M+50K	*n*=120	1.46	9.38	41.20	47.96
	*n*=180	0.08	1.68	20.06	78.18
	*n*=60	33.04	31.94	27.78	7.24
10M-10M+50K	*n*=120	1.52	9.34	41.32	47.82
	*n*=180	0.10	1.48	19.44	78.98
	*n*=60	31.92	32.64	28.00	7.44
15M-15M+50K	*n*=120	1.40	8.36	41.90	48.34
	*n*=180	0.08	1.42	21.20	77.30
	*n*=60	31.82	32.04	28.36	7.78
25M-25M+50K	*n*=120	1.48	9.32	40.86	48.34
	*n*=180	0.02	1.38	20.40	78.20
	*n*=60	32.06	32.44	27.88	7.62
32.5M-32.5M+50K	*n*=120	1.42	9.44	40.50	48.64
	*n*=180	0.06	1.32	19.90	78.72
	*n*=60	33.18	31.84	27.20	7.78
40M-40M+50K	*n*=120	1.48	9.38	41.04	48.10
	*n*=180	0.12	1.60	20.12	78.16
	*n*=60	31.90	31.94	29.42	6.74
50M-50M+50K	*n*=120	1.54	8.76	41.08	48.62
	*n*=180	0.02	1.48	20.72	77.78
	*n*=60	32.58	31.10	28.38	7.94
75M-75M+50K	*n*=120	1.22	8.78	39.94	50.06
	*n*=180	0.02	1.34	19.84	78.80
	*n*=60	31.40	32.66	28.90	7.04
100M-100M+50K	*n*=120	1.50	9.08	40.38	49.04
	*n*=180	0.08	1.36	20.02	78.54

**Table 6:** **Percentage of the NGS-reads experiments recovering certain number of global markers after feature selection for COV=0.1 and** ***ρ*** **=0** ***.*** **8**

**NGS-reads**	**Sample**	**<8**	**8**	**9**	**10**
**range**	**size**	**markers**	**markers**	**markers**	**markers**
	*n*=60	99.40	0.52	0.08	0.00
1K-50K	*n*=120	68.14	19.18	10.92	1.76
	*n*=180	29.54	28.16	30.44	11.86
	*n*=60	73.14	16.55	8.57	1.74
50K-100K (SAGE-tags)	*n*=120	14.16	21.13	36.43	28.28
	*n*=180	2.52	8.03	30.16	59.29
	*n*=60	44.26	21.56	23.10	11.08
250K-300K	*n*=120	5.54	10.34	29.88	54.24
	*n*=180	0.84	3.06	15.92	80.18
	*n*=60	36.74	22.00	26.16	15.10
500K-550K	*n*=120	4.74	9.10	28.14	58.02
	*n*=180	0.56	2.66	13.68	83.10
	*n*=60	32.76	20.90	26.96	19.38
5M-5M+50K	*n*=120	4.38	7.30	24.74	63.58
	*n*=180	0.62	2.08	11.92	85.38
	*n*=60	32.56	20.06	26.84	20.54
10M-10M+50K	*n*=120	4.38	8.72	23.72	63.18
	*n*=180	0.70	2.18	11.62	85.50
	*n*=60	31.90	21.54	27.70	18.86
15M-15M+50K	*n*=120	4.46	8.16	23.28	64.10
	*n*=180	0.76	2.12	12.02	85.10
	*n*=60	32.56	21.02	26.90	19.52
25M-25M+50K	*n*=120	3.96	8.44	24.30	63.30
	*n*=180	0.54	2.32	11.86	85.28
	*n*=60	33.00	21.30	26.74	18.96
32.5M-32.5M+50K	*n*=120	4.18	7.20	24.10	64.52
	*n*=180	0.64	1.98	10.94	86.44
	*n*=60	32.94	21.00	27.26	18.80
40M-40M+50K	*n*=120	4.36	7.48	24.22	63.94
	*n*=180	0.80	2.24	11.16	85.80
	*n*=60	32.70	20.64	26.70	19.96
50M-50M+50K	*n*=120	4.14	8.52	23.42	63.92
	*n*=180	0.74	2.32	11.84	85.10
	*n*=60	33.38	20.34	27.02	19.26
75M-75M+50K	*n*=120	4.48	7.90	24.26	63.36
	*n*=180	0.60	1.92	11.00	86.48
	*n*=60	32.70	21.34	26.86	19.10
100M-100M+50K	*n*=120	4.54	7.88	23.64	63.94
	*n*=180	0.80	2.58	11.64	84.98

The true error of a designed classifier measures the generalization capability of the classifier on a future sample point. Given a set of training sample points and a classification rule, one needs the full feature-label probability distribution to calculate the true error of the classifier designed on the training set. In this study, we find the true error of a classifier with a large independent sample. Because the training sample is random, the true error is a random variable with its own probability distribution. Therefore, to demonstrate the actual performance of the designed classifiers, the estimated probability density function (PDF) of the true error for each classification rule, distribution model and all data generation models (MVN-GC, NGS-reads and SAGE-tags) is presented.

**Figure 3:**
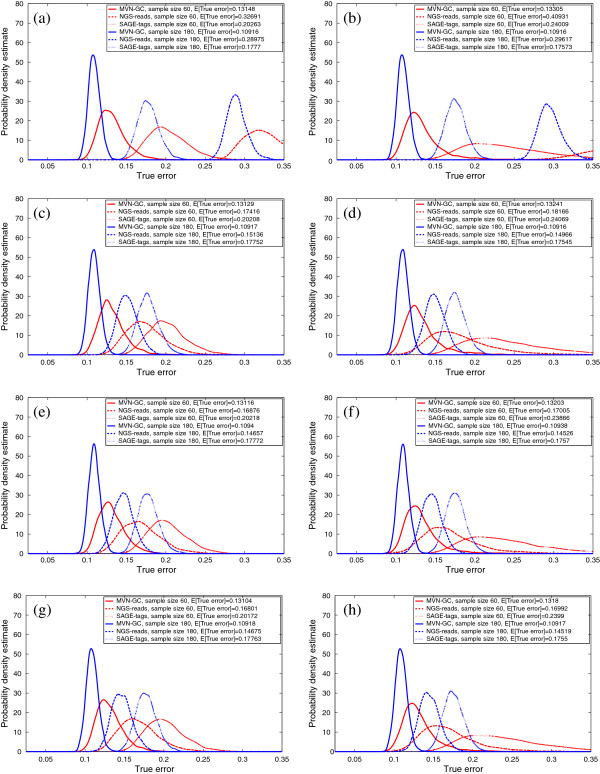
**Probability density estimate of the true error for LDA classification rule, without feature selection (left column,** ***d*** **=10 global markers are directly used), with** ***t*-test feature selection (right column),** ***ρ*** **=0** ***.*** **8, COV =0** ***.*** **05,**$\underset{j}{\sum }X_{i,j}\in (50K,100K)$** for SAGE-tags and the following total number of reads for NGS-reads: (a,b)**$\underset{j}{\sum }X_{i,j}\in (1K,50K)$**; (c,d)**$\underset{j}{\sum }X_{i,j}\in (250K,300K)$**; (e,f)**$\underset{j}{\sum }X_{i,j}\in (25M,25M+50K)$**; (g,h)**$\underset{j}{\sum }X_{i,j}\in (50M,50M+50K)$**.**

**Figure 4:**
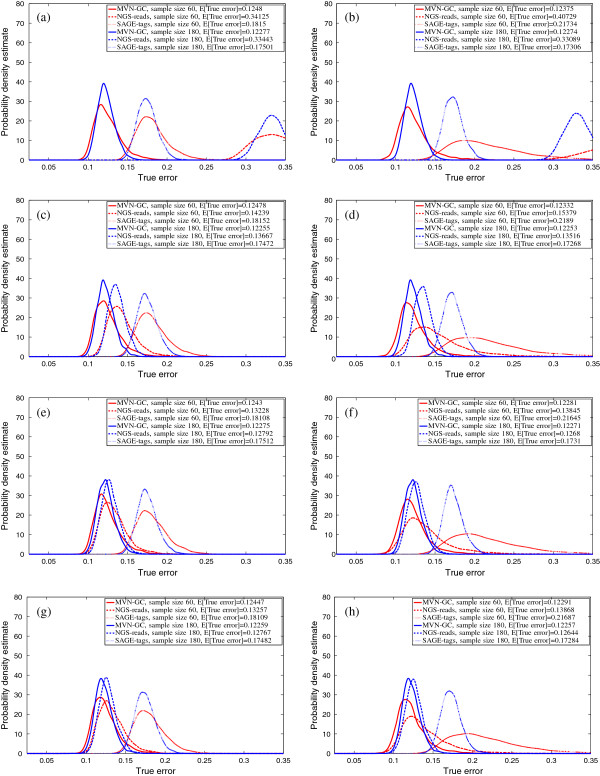
**Probability density estimate of the true error for 3NN classification rule, without feature selection (left column,** ***d*** **=10 global markers are directly used), with** ***t*-test feature selection (right column),** ***ρ*** **=0.8, COV =0.05,**$\underset{j}{\sum }X_{i,j}\in (50K,100K)$** for SAGE-tags and the following total number of reads for NGS-reads: (a,b)**$\underset{j}{\sum }X_{i,j}\in (1K,50K)$**; (c,d)**$\underset{j}{\sum }X_{i,j}\in (250K,300K)$**; (e,f)**$\underset{j}{\sum }X_{i,j}\in (25M,25M+50K)$**; (g,h)**$\underset{j}{\sum }X_{i,j}\in (50M,50M+50K)$**.**

**Figure 5:**
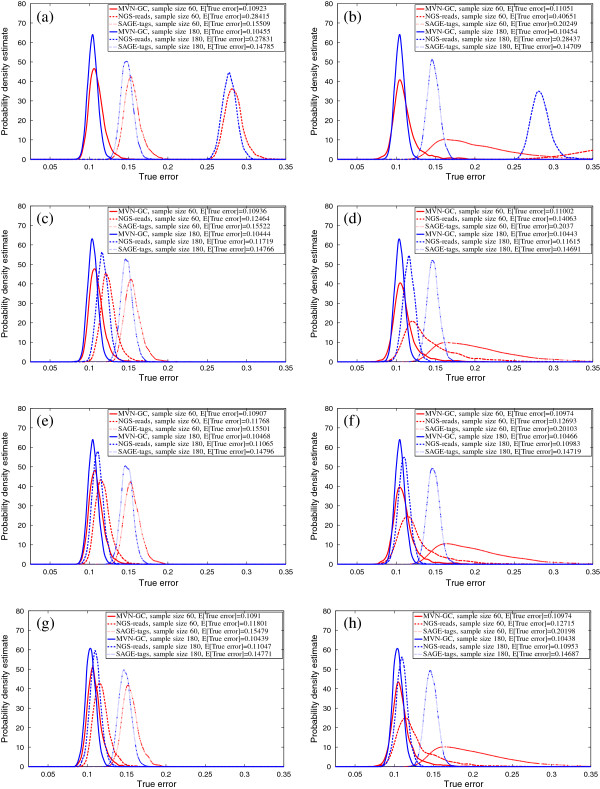
**Probability density estimate of the true error for RBF-SVM classification rule, without feature selection (left column,** ***d*** **=10 global markers are directly used), with** ***t*-test feature selection (right column),** ***ρ*** **=0.8, COV =0.05,**$\underset{j}{\sum }X_{i,j}\in (50K,100K)$** for SAGE-tags and the following total number of reads for NGS-reads: (a,b)**$\underset{j}{\sum }X_{i,j}\in (1K,50K)$**; (c,d)**$\underset{j}{\sum }X_{i,j}\in (250K,300K)$**; (e,f)**$\underset{j}{\sum }X_{i,j}\in (25M,25M+50K)$**; (g,h)**$\underset{j}{\sum }X_{i,j}\in (50M,50M+50K)$**.**

We report the true errors of the designed classifiers for two different feature-selection schemes and classification rules. In the first scheme, no feature selection is performed and the first *d* global markers are directly used for classification. In the second scheme, *t*-test feature selection is done before a classifier is designed to select the best *d* features. Figures 3, 4 and 5 show the salient finding of this study. We present the PDF of the true error for different classification rules trained on 60 and 180 sample points when the correlation among features in the same block is high (*ρ* = 0.8) and COV = 0.05. Distributions with higher and tighter densities around lower true errors indicate better classifier performance. If the PDFs can be approximated as univariate Gaussians, then a good classification performance amounts to smaller mean and variance. In all cases, for similar sample sizes and similar settings for *ρ* and COV, MVN-GC outperforms the two other data types. This is not surprising since it is considered as the ground truth. Also, it is evident that a larger sample size gives better classifiers with smaller variance as illustrated by the distribution of the true error.

Figures 3, 4 and 5 show that utilizing the best *d* features (with or without feature selection) in the model, SAGE-tags and NGS-reads for small ranges of the total reads yield similar results (or even much worse when the range is smaller) in terms of the classification performance, and both are inferior when compared to the ground truth. This may lead to a conclusion that one should not expect improvements in classification performance when gene-expression levels are processed through an NGS-reads pipeline. However, our main objective here is to show that as one increases the total number of reads for the NGS-reads model, improvement can be achieved and the error rates decrease. This conclusion confirms the intuition provided by a simple calculation about the increase of the separability of two Poisson random variables with means proportional to the number of reads. However, notice that the modeling of the sequencing pipeline introduces randomness in the means of the respective Poisson parameters describing the individual gene reads. Moreover, our focus is not that much on the separability of the two classes/phenotypes but rather on the classification performance as measured by the classification error and its dependence on ground truth (MVN-GC model) sample size, sequencing depth, and feature vectors. Our goal for modeling NGS-reads with small ranges is to demonstrate the performance of SAGE and RNA-Seq, when their data is similar. This result suggests that having a larger number of reads for the RNA-Seq experiments could compensate for the errors that can occur during the NGS-reads pipeline sample processing. Here we have shown the results only for COV = 0.05, since in our observations, it appears that changing COV has little effect on the distribution of the true error for both SAGE-tags and NGS-reads models, except that it slightly increases the variance of the PDFs. The effect of feature selection (right columns) on the performance is best shown when the sample size is small. In this case, the variance of the true error is so large that drawing any conclusion regarding the performance on a small sample would be futile. Another interesting observation is that, on average, 3NN and RBF-SVM classification rules outperform LDA for both NGS-reads and SAGE-tags data, supporting the idea that using linear classifiers for these types of data is not the best way to proceed in a highly non-Gaussian model – as is the case after the data have gone through the pipeline. The best rates for the expected true error across all settings are achieved by the RBF-SVM classification rule, even for small samples, especially with small variance.

## Conclusions

In this paper, we model gene-expression levels as a multivariate Gaussian distribution that statistically captures the real mRNA levels within the cells. The newly developed technologies of sequencing DNA/RNA, referred to as NGS, and their ascendant SAGE technology generate discrete measures for gene expressions. The count data from these technologies can be modeled with a Poisson distribution. The multivariate Gaussian gene expressions are transformed through a Poisson filter to model NGS and SAGE technologies. The three categories of data are subjected to feature selection and classification. The objective is to evaluate the performance of the NGS technologies in classification. Our simulations show that when the gene expressions are directly used in classification, the best performance in terms of the classification error is achieved. The NGS-reads model generates considerably higher coverage for genes and can outperform SAGE in classification, when the experiment generates large number of reads. Even though NGS still has a variety of error sources involved in its process, its high volume of reads for a specific gene can lower the chance of misclassification. Thus, it is important to use the highest possible coverage for the entire genome while performing a RNA-Seq analysis if the goal of the study is to distinguish the samples of interest. Nevertheless, one must recognize that, as is typical with nonlinear transformations, the NGS pipeline transforms the original Gaussian data in such a way as to increase classification difficulty. As more refined modeling becomes available for the NGS pipeline, further study needs to performed, as has been done in the case of the LC-MS proteomics pipeline [1], to determine which segments of the pipeline are most detrimental to classification and what, if anything, can be done to mitigate classification degradation.

## Competing interests

The authors declare that they have no competing interests.

## Authors’ contributions

NG proposed the main idea, planned/structured the study, and worked on the simulations and manuscript. MRY contributed to the main idea, simulation, structure of the study, and the manuscript. CDJ and II helped in conceiving the study and revised the manuscript. ERD contributed to the formulation of main idea and also to the steps of the study, and revised the manuscript. All authors read and approved the final manuscript.
